# Development of a Mouse Model to Explore CD4 T Cell Specificity, Phenotype, and Recruitment to the Lung after Influenza B Infection

**DOI:** 10.3390/pathogens11020251

**Published:** 2022-02-15

**Authors:** Ajitanuj Rattan, Chantelle L. White, Sean Nelson, Max Eismann, Herbey Padilla-Quirarte, Maryah A. Glover, Thamotharampillai Dileepan, Bindumadhav M. Marathe, Elena A. Govorkova, Richard J. Webby, Katherine A. Richards, Andrea J. Sant

**Affiliations:** 1 David H. Smith Center for Vaccine Biology and Immunology, Department of Microbiology and Immunology, University of Rochester Medical Center, Rochester, NY 14642, USA; ajitanuj.rattan@gmail.com (A.R.); chantelle_white@urmc.rochester.edu (C.L.W.); sean_nelson@urmc.rochester.edu (S.N.); max_eismann@urmc.rochester.edu (M.E.); maryah_glover@urmc.rochester.edu (M.A.G.); katherine_skelly@urmc.rochester.edu (K.A.R.); 2 Department of Microbiology and Immunology, Emory University School of Medicine, Atlanta, GA 30322, USA; herbey.o.padilla-quirarte@emory.edu; 3 Department of Microbiology and Immunology, University of Minnesota Medical School, Minneapolis, MN 55455, USA; dile0003@umn.edu; 4 Department of Infectious Diseases, St. Jude Children’s Research Hospital, Memphis, TN 38105, USA; bindumadhav.marathe@stjude.org (B.M.M.); elena.govorkova@stjude.org (E.A.G.); richard.webby@stjude.org (R.J.W.); 5 Center for Influenza Disease and Emergence Response (CIDER), University of Rochester Medical Center, Rochester, NY 14642, USA

**Keywords:** influenza virus, immune response, CD4 T cell

## Abstract

The adaptive T cell response to influenza B virus is understudied, relative to influenza A virus, for which there has been considerable attention and progress for many decades. Here, we have developed and utilized the C57BL/6 mouse model of intranasal infection with influenza B (B/Brisbane/60/2008) virus and, using an iterative peptide discovery strategy, have identified a series of robustly elicited individual CD4 T cell peptide specificities. The CD4 T cell repertoire encompassed at least eleven major epitopes distributed across hemagglutinin, nucleoprotein, neuraminidase, and non-structural protein 1 and are readily detected in the draining lymph node, spleen, and lung. Within the lung, the CD4 T cells are localized to both lung vasculature and tissue but are highly enriched in the lung tissue after infection. When studied by flow cytometry and MHC class II: peptide tetramers, CD4 T cells express prototypical markers of tissue residency including CD69, CD103, and high surface levels of CD11a. Collectively, our studies will enable more sophisticated analyses of influenza B virus infection, where the fate and function of the influenza B-specific CD4 T cells elicited by infection and vaccination can be studied as well as the impact of anti-viral reagents and candidate vaccines on the abundance, functionality, and localization of the elicited CD4 T cells.

## 1. Introduction

Animal models of influenza infection and vaccination are tremendously valuable for exploring issues related to influenza virus biology, transmission, immunity to infection, and to the development of protective vaccines. These models have been used to characterize the kinetics of infection and viral clearance, elicitation of innate and adaptive responses, testing of antiviral compounds, and for preclinical studies of vaccine candidates. Different animal models, including the ferret, pigs, hamsters, guinea pigs, mice, and non-human primates have inherent strengths and weaknesses that have been discussed in several recent reviews [1,2,3,4,5]. Animal models also permit invasive sampling of tissues, such as lung and lymph nodes, in order to analyze events such as the germinal center response and tissue-resident immunity. These advantages provide key mechanistic insight into the events associated with protective immunity to infection and into vaccine responses.

The most widely used immunology model for influenza virus is the mouse, which has a number of notable advantages that are not present in other models of infection. First, inbred mouse strains are readily available and economically feasible to house and study in large cohorts, allowing reproducibility and well-controlled analyses of the effect of age, sex, and other comorbidities on disease susceptibility and vaccine responses [6,7,8,9,10,11,12]. Importantly, mice also enable highly sophisticated analyses of the impact of genetic polymorphisms in susceptibility and resistance to infection through the use of different inbred and congenic mice [13,14,15] and through more recent developments such as the collaborative cross strains of mice [16,17]. Inbred strains of mice with well-characterized MHC class I and class II proteins enable epitope-specific tracking of T cell responses with synthetic peptides and derivation of MHC-peptide multimers that allow detailed characterization of the vaccine- or infection-elicited CD4 and CD8 T cells. Finally, an exceptional advantage of mice for understanding immunity to influenza is the availability of genetic tools such as traditional transgenic mice and conditional knock-out or knock-in strains of mice. These genetic tools are complemented by a wide array of well-characterized antibodies that selectively react with markers on lymphoid cell subsets in both the innate and adaptive compartments, cytokines, and chemokines that can be used to either detect or perturb the participation of cells or mediators in the immune response.

Of particular value in dissecting the events in immunity to influenza B (IBV) is the advantages offered by C57BL/6 (B6) mice [18,19]. These mice offer a wide and diverse array as well as an availability of commercially available genetic models that perturb events in the developing immune response to infection. Examples include gene-deficient animals that eliminate critical cell types or mediators (e.g., [20,21,22,23,24]). In addition, the B6 strain of mice, expressing a single MHC class II molecule (I-A^b^), enables the ready production of peptide: MHC tetramers [23,25,26,27,28]. When coupled with knowledge of epitopes recognized by CD4 T cells for IBV, these genetic strategies will enable a significantly enhanced understanding of the regulation of protective immunity to IBV. These strategies have been used extensively to study the CD8 T cell responses to influenza A infection (IAV) in mice and their localization, in vivo trafficking, T cell repertoires, and fate after infection [29,30,31,32,33,34,35,36,37]. Finally, these advantages of B6 mice are complemented by the use of recent human isolates of IAV and IBV, as we report here. These viruses provide an advantage to much older mouse-adapted strains of virus because the genetic features of the influenza virus strains have not been extensively perturbed by extended passage in mice as mouse-adapted viruses often are [21,38].

There is a great need for more mechanistic insight into our understanding of infection and vaccine responses. Epidemiological studies of influenza B in human populations have now provided clear evidence for the morbidity and mortality associated with influenza B, particularly in young children (reviewed in [39,40,41,42]), but there has been little insight into the unique features of immunity to infection by these viruses. There have been only a small number of published experimental studies on the adaptive T cell response to influenza B infection and vaccination [43,44] and are particularly limited with regard to the CD4 T cell compartment. CD4 T cells have a number of key effector functions that promote immunity to influenza virus. Although sterilizing immunity to influenza is provided by neutralizing antibodies [45,46,47,48], the development of high affinity, class-switched antibodies is dependent on follicular helper cells, which are a subset of CD4 T cells elicited by both infection and vaccination (reviewed in [49,50,51,52]). Influenza-specific CD4 T cells also play key additional roles in the immune response to influenza (reviewed in [53,54,55]). They are critical for protective immunity conveyed by cytotoxic CD8 T cells, enhancing their priming, expansion, and establishment of long-lived memory. CD4 T cells elicited by infection also promote the recruitment of innate effectors to the lung, blunting virus replication, and they can also provide protection by their cytotoxic activity that has the potential to directly eliminate infected cells (reviewed in [56,57,58,59]). Although many of these functions of CD4 T cells have been explored for decades for influenza A viruses, there are almost no data on the specific contributions of CD4 T cells in response to IBV infection, nor tracking of the IBV-elicited epitope specific CD4 T cells.

In this study, we have used experimental infection of B6 mice with a commonly studied Victoria strain of influenza (B/Brisbane 60/2008) to develop a valuable experimental model system to analyze the CD4 T cell responses to infection. Using an iterative peptide discovery strategy, we have identified immunodominant CD4 T cell epitopes from major proteins expressed by this virus. Using this information, we have analyzed the localization of infection-elicited virus-specific CD4 T cells in secondary lymphoid tissues and lung, and we have examined the distribution of influenza B-specific CD4 T cells in lung sub-compartments. We have also evaluated the production of anti-viral cytokines and expression of cell surface proteins associated with tissues residence. These studies lay the groundwork for future mechanistic studies of the unique features of influenza B infection, evaluation of anti-viral mediators, and preclinical studies of vaccine responses.

## 2. Results

### 2.1. Infection and Viral Replication of Influenza B after Infection of B6 Mice

**Figure 1:**
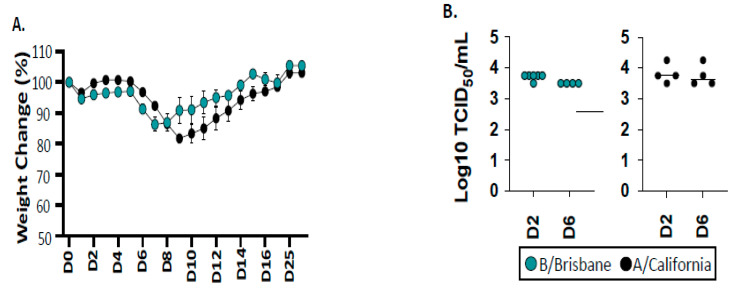
Infection, weight loss, and viral titers after infection of C57BL/6 mice. Panel (**A**) shows the weight loss curves for IBV (turquoise) and IAV (black). A group of female mice (≈8–12 weeks old) were anesthetized and infected intranasally either with IAV or IBV, diluted in 30 μL of phosphate-buffered saline (PBS). After infection, the mice were weighed individually each day and observed for signs of illness and mortality at the days post infection (D). Results are represented as the mean with SEM for individual mice pooled from two independent experiments. Body weight at time of infection was set to 100%. No statistically significant differences in weight loss were detected between the two cohorts (*t*-test, *p* > 0.05). Panel (**B**) illustrates the TCID50 to quantifying the viral lung titers at two time points post-infection (day 2 and day 6), as indicated.

To gain insight into the adaptive T cell response to influenza B, we established conditions to infect the widely used B6 mouse strain with influenza B/Brisbane/60/2008 (IBV), which is a Victoria lineage strain that has been previously described [60]. We examined influenza A/California/04/2009 (IAV), an H1N1 virus that emerged in humans in 2009 in parallel, which we have previous studied in mouse models of infection [61,62]. These strains of recent human isolates of influenza virus have not been modified by adaptation in mice, unlike other commonly used IAV viruses such as the H1N1 A/Puerto Rico/8/34 (PR8) or A/HKx31 (X31) H3N2 viruses. Infection doses for these viruses were identified for these studies in B6 mice, based on evidence of viral replication, as well as similar viral titers early in the response and, finally, that elicited virus-specific T cells. Shown in Figure 1 is an example of weight loss assayed throughout the course of infection and lung viral titers measured early after infection. At the doses used, there is approximately 15–20% weight loss that peaks at day 8–9 for both viruses, from which the mice recover by day 10–14. This, coupled with the viral titers shown in Figure 1B, provides clear evidence of virus replication.

### 2.2. The Specificity, Abundance, and Localization of IBV-Specific CD4 T Cells

**Figure 2:**
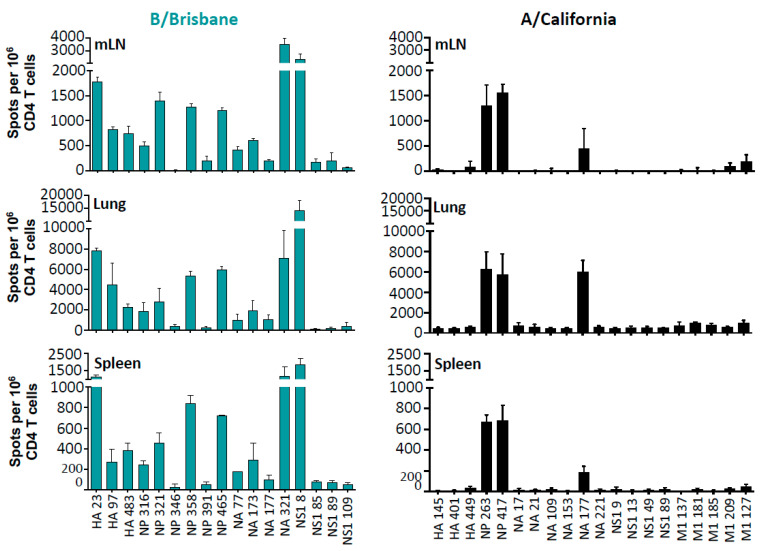
CD4 T cell epitope specificity and diversity after infection. IFNγ EliSpot assays were used to identify immunodominant CD4 T cell epitopes to individual viral proteins of IBV (**left**) and IAV (**right**) 10 days post-infection. Individual peptides were used to stimulate MACS-enriched CD4 T cells from mediastinal lymph nodes (mLN), lung and spleen (top, middle, and bottom panels, respectively). Results are presented as the mean number of spots per million CD4 T cells ± SEM for individual peptides from three independent experiments with background subtracted.

After establishing experimental conditions to infect the B6 mice, we evaluated the kinetics of recruitment of CD4 T cells so that we could assess elicitation of CD4 T cells at the peak of the response, and we found that day 10–11 post infection was the optimal point to sample tissues for the isolation of CD4 T cells (see Supplemental Table S2). We first used overlapping peptide pools that represented the entire translated sequence of the major structural proteins of IBV and IAV (not shown) to assess the elicitation of the CD4 T cell repertoire to IBV in conjunction with cytokine EliSpots, using CD4 T cells that were enriched by negative bead isolation. An iterative process of screening was performed with small pools of individual peptides, using a strategy originally described by Tobery for CD8 T cells [63] and described previously by our laboratory to identify the IAV candidate major CD4 T cell epitopes [64,65,66]. Three tissues sampled post-infection (lung, mediastinal lymph node (mLN), and spleen) were used to assess CD4 T cell reactivity, which include both the site of infection and peripheral lymphoid tissue. In Figure 2, the single peptide epitopes identified are indicated from the mLN, the lung tissue, and spleen. These assays revealed, first, that influenza-specific CD4 T cells are detectable in all three tissues sampled: mLN, spleen, and respiratory tract, indicating dispersal into both secondary lymphoid tissue and recruitment into the tissue site of infection. Second, these results indicate that IBV elicits CD4 T cells of very broad antigen specificity that includes major epitopes from HA, NP, NA, and NS1. As a non-structural protein, NS1 is largely absent from virions, and the epitope mapping indicates that CD4 T cells specific for four distinct NS1 peptides (one major and three minor) were elicited from IBV infection and detectable in lung, mLN, and spleen. All peptide specificities were also seen when IL-2 producing cells were quantified (data not shown). The elicitation of robust NS1 CD4 T cell reactivity further demonstrates the active infection of IBV within the B6 mice. In contrast to IBV, IAV infection in B6 mice displayed a more restricted CD4 T cell repertoire, which was primarily specific for two dominant peptide epitopes in NP and one in NA. We defined epitope-specific CD4 T cells as “dominant” if they recruited approximately 500 cytokine-producing cells per million CD4 T cells in the mLN, 2000 cells per million in the lung, and 250-cytokine producing cells in the spleen at the peak of the response. Similar patterns of immunodominance and distribution of CD4 T cells were observed in both male and female mice (data not shown), indicating that sex does not detectably influence the factors involved in epitope selection or distribution across tissues in vivo.

### 2.3. MHC-Dependent Viral Protein Biases in CD4 T Cell Specificity

The striking enrichment of CD4 T cells specific for HA epitopes in the IBV-infected mice raised the issue of whether IBV has a higher absolute abundance of HA available to elicit CD4 T cells, relative to IAV. We also considered whether specific features of post-translational modification on HA-B, derived from IBV Brisbane [67,68], enhances its handling by APC and thus its immunogenicity or interactions with lung surfactants, as has been shown with IAV [69,70,71]. To address these issues, we explored the pattern of CD4 T cell immunodominance in two MHC congenic mouse strains that differ only in the sequence of the MHC-encoded proteins that they express and thus the peptides that they present to CD4 T cells. Our previous studies with an alternate influenza virus (A/New Caledonia/20/99) suggested that I-A^b^, expressed in B6 and B10 mice, tends to elicit a narrower repertoire of CD4 T cells, while I-A^s^, expressed in the B10 congenic strain B10.S, is able to present a more diverse set of peptides [65,72]. These congenic mice share all of the background non-MHC genes, but they are expected to present a distinct set of peptides for the recruitment of CD4 T cells. The use of these two strains gave us the potential to assess whether the HA-B protein was in general more highly immunogenic than HA-A (influenza hemagglutinin derived from H1N1) after infection with IBV vs. IAV, respectively.

**Figure 3:**
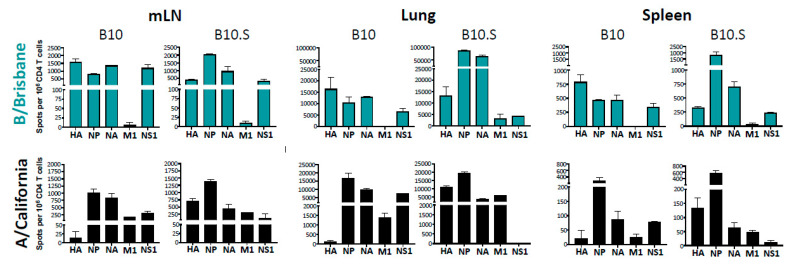
MHC-dependent selection of CD4 T cell viral epitopes. B10 or B10.S mice were infected with IBV (**top**) or IAV (**bottom**), and at day 11 post-infection, CD4-enriched populations of cells were isolated from mLN, lung, or spleen of infected mice and were tested for reactivity to the indicated viral proteins using pools of overlapping peptides, representing the entire translated sequence of each protein. CD4 T cells reactive to the peptide pools were quantified by IFNγ EliSpots. Results are presented as the average spot count per million CD4 T cells with the range shown.

The B10 and B10.S mice were infected with a non-lethal dose of influenza, and at day 11, their lung, mLN, and spleen were sampled. Since individual epitopes were not identified for the A/California strain of IAV or IBV in B10.S mice, the viral protein specificity of the CD4 T cells was assessed through the use of entire peptide pools for HA, NA, NP, M1, and NS1. As before, cytokine EliSpots were used to quantify CD4 T cell reactivity to the viral proteins. The results of these experiments are shown in Figure 3. B10 mice exhibited a pattern of CD4 T cell reactivity very similar to B6, as expected. The majority of CD4 T cells specific for IBV in B10 mice were specific for HA, NA, NP, and NS1, while for IAV, the major epitopes were derived from NP, NA, M1, and NS1, which is similar to that observed in B6 mice. When B10.S mice, expressing the alternate MHC class II I-A^s^ molecule, were sampled post infection, and CD4 T cells were purified and tested, reactivity to HA-A, NP, NA, and NS1 was apparent. These CD4 T cells are likely specific for different individual peptides than in B10 mice [65] because of the MHC class II allele-dependent presentation of peptides. In B10.S mice, robust CD4 T cell reactivity to HA-A was apparent and in fact was greater in abundance than that detected for IBV. We conclude from this set of experiments that the dominant feature in determining viral antigen specificity in CD4 T cells after influenza A or influenza B infection was the selecting MHC class II allelic protein rather than generalized features of protein abundance or antigen handling of HA antigens expressed in IBV and IAV.

### 2.4. Distribution of Cytokine-Producing IBV-Specific CD4 T Cells after Infection

**Figure 4:**
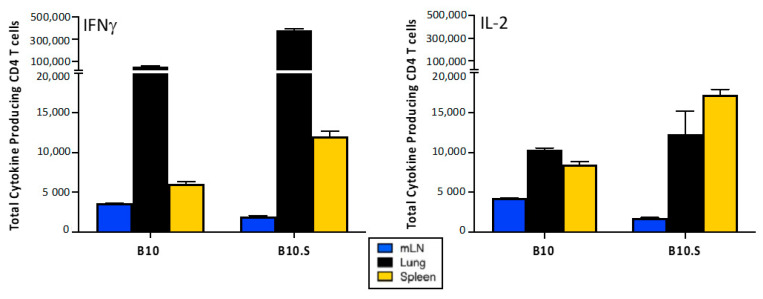
Tissue distribution of influenza B reactive CD4 T cells at the peak of the response to B/Brisbane infection. B10 and B10.S mice (*n* = 4 per strain) were intranasally infected with 3840 PFU of B/Brisbane/60/08. At 11 days post-infection, the mediastinal lymph node (mLN) (blue), lung (black), and spleen (gold) were harvested. Cytokine EliSpots were performed in which CD4 T cells were purified from single cells suspensions and stimulated with influenza B HA, NP, NA, M1, and NS1 peptide pools. Influenza B reactivity was determined by IFNγ (**left**) and IL-2 (**right**) secretion. The total response to the peptide pools for each tissue was summed, and the data are presented as the total number of cytokine-producing CD4 T cells per mouse in each tissue, with the range of response shown.

In order to quantify the distribution of IBV-specific CD4 T cells in the three compartments sampled (mLN, lung, and spleen), the number of viral antigen-specific EliSpots detected using IFN-γ and IL-2 for all viral antigen reactivities were summed. Then, this frequency of virus-specific CD4 T cells was back calculated to the yield of CD4 T cells isolated from each tissue. Then, these values, as shown in Figure 4, were represented to illustrate the percentage of total virus specific cytokine-producing cells in each tissue. These data demonstrate that IFN-γ (left)-producing cells are preferentially recruited to/accumulate in the lung, while almost half of the IL-2 (right)-producing cells accumulate in the spleen. By both measures to quantify CD4 T cells, IBV-specific cells are seen to be distributed across the animal at a peak time (day 11) after infection and include both the site of infection and the peripheral lymphoid tissues.

### 2.5. Distribution and Cell Surface Phenotype of IBV-Specific CD4 T Cells in the Lung

**Figure 5:**
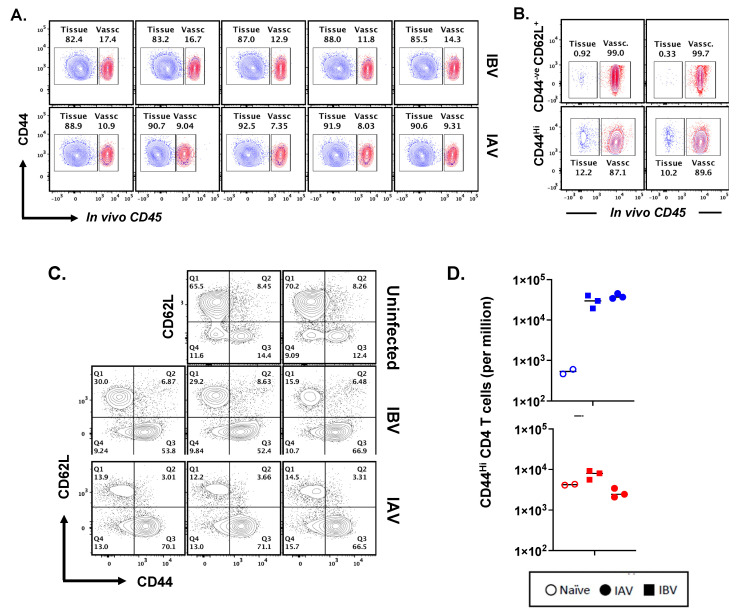
Distribution of virus-specific CD4 T cells into lung tissue and vasculature. Just prior to sacrifice, mice infected with IBV (top) or IAV (bottom) were injected IV with APC-labeled CD45 to label cells in the lung vasculature. Shown in (**A**) are the flow cytometry patterns of individual mice that illustrate the distribution of CD44hi CD4 T cells in lung tissue (in blue, unlabeled cells) and vasculature (in red, labeled cells), showing that approximately 83–91% of the CD4 T cells are in the lung tissue. Panel (**B**) shows the distribution of CD4 T cells from lungs of two individual naïve mice defined as naïve (CD44-ve, CD62L+, top panels) or antigen-experienced (CD44hi, bottom panels) after IV labeling to identify vascular localized cells (in red, labeled) or tissue (in blue, unlabeled). Panel (**C**) illustrates the percent of CD44Hi and Naïve CD4 (CD44- CD62L+) T cells in the lung of uninfected (top), IBV infected (middle), or IAV infected mice (bottom). Panel (**D**) is the quantification of cells in the vasculature (red) or tissue (blue) from naïve (open symbols), IAV-infected (circles), or IBV-infected mice (squares). These data illustrate that although the CD44hi CD4 cells in the vasculature are not enriched after infection, the CD44hi, CD4 T cells in the tissue have increased approximately 50–100 fold at day 10 post-infection. The gating strategy is shown in Supplementary Figure S1.

After having established that CD4 T cells of diverse antigen specificity are elicited by IBV and that many of the elicited CD4 T cells are recruited back into the lung post-infection, we sought to examine the localization of the elicited CD4 T cells within the lung compartments. For the peptide-stimulated cytokine EliSpot assays presented thus far, the CD4 T cells were isolated from the entire lung tissue, and thus, the sub-compartment of localization was not established. The lung is a highly vascularized organ and is enriched with a network of blood vessels that serves to promote the trafficking and access of elicited innate and adaptive components of the immune system into the lung tissue. Intravascular labeling (IV) in vivo is now a well-characterized strategy for the clear discrimination of immune cells localized in the pulmonary vasculature and tissue [73], with the latter term comprising the airways, parenchyma, and interstitium. Although primarily explored for CD8 T cells [74,75,76,77,78], recent studies by our group [79,80] and others [78,81,82,83,84,85] have shown that influenza-specific CD4 T cells home to lung tissue to deliver anti-viral effector function. To examine the localization of CD4 T cells elicited by IBV in lung compartments, we utilized the IV-labeling strategy employed by others. Prior to the sacrifice of infected mice, they were administered a fluorescently labeled antibody, specific for CD45, intravenously for 3–4 min, to label all hematopoietic cells in the vasculature, while preserving cells sequestered in tissue in an unlabeled state. This procedure allows the clear demarcation, quantification, and characterization of the cells in the lung vasculature relative to the lung tissue. Figure 5A shows flow cytometry profiles of the lung cells in replicate animals infected with either IBV or IAV (see Supplemental Figure S1 for gating scheme) with evident distinction of IV positive (in red) and IV negative (in blue), in antigen-experienced cells, identified by high expression of CD44 [79,81]. When distinguished by IV labeling, most (85–90%) of the virus-specific lung-localized CD4 T cells are within the lung tissue rather than the lung vasculature for both IBV (top) and IAV (bottom), and they are distinct from that in naïve mice (Figure 5B) where most of the CD4 T cells are in the vasculature. To examine the distribution and abundance of CD4 T cells in the lungs of naïve mice, multiparameter flow cytometry was used to identify both naïve CD4 T cells and antigen-experienced CD4 cells using CD62L and CD44 (Figure 5C). These studies show antigen-experienced influenza-elicited CD4 T cells, expressing high levels of CD44, gain access to and accumulate substantially in the lung tissue of infected but not naïve mice. Thus, the strategy of using the surface expression of CD44 and CD11a allows us to define the features of the polyclonal CD4 T cell responses to infection. We have also found that influenza-specific, cytokine-producing CD4 T cells are recovered from the BAL in IBV-infected mice (data not shown). The frequency of CD4 T cells in lung vs. BAL is similar between IAV and IBV; however, the lung tissue contains greater than 50 times more CD4 T cells than that recovered in the BAL. These data indicate that the total number of influenza-elicited CD4 cells is much higher in the lung parenchyma in response to both strains of influenza at the peak of the response to infection.

**Figure 6:**
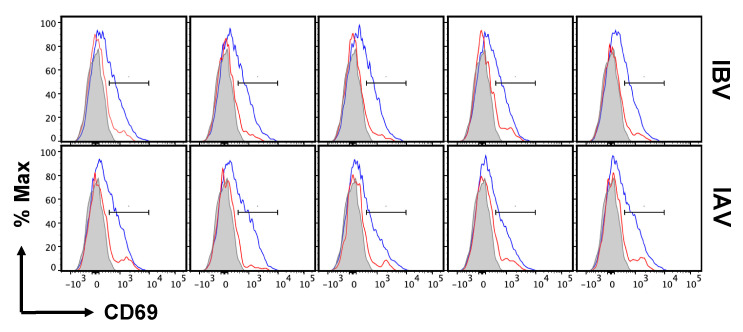
Expression of CD69 on tissue-resident CD4 T cells elicited by influenza infection. Flow histograms for six individual mice showing the expression of CD69+ CD44hi CD4 T cells localized to either lung tissue (blue) or lung vasculature (red) identified as described in Figure 5. Shown are the patterns from CD4, CD44hi cells from IBV (top) and IAV (bottom) infected mice. The gray profile represents the FMO control.

**Figure 7:**
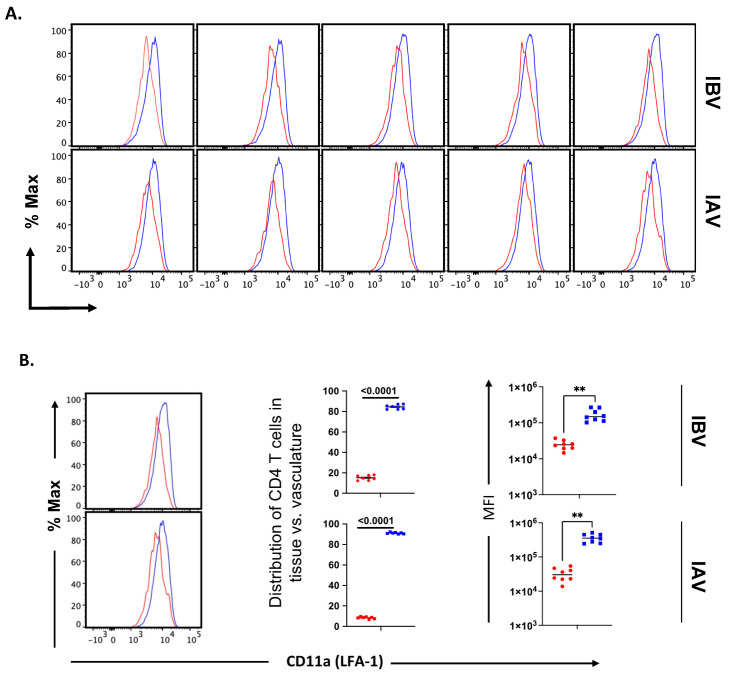
Expression of CD11a by lung vasculature and lung tissue CD4 T cells after IBV and IAV infection. (**A**) Flow cytometry profiles of CD11a expression in Cd44hi CD4 T cells from the lungs of individual mice infected IBV (top) or IAV (bottom) identified in lung tissue (blue) or lung vasculature (red). (**B**) Independent examples of CD11a expression shown at right and statistical treatment from data from multiple mice as the percentage of CD4 T cells in lung tissue vs. vasculature that express both high levels of CD11a and CD44 from individual mice is shown in the middle panel. The surface levels of CD11a (MFI) in lung vasculature or tissue in CD4 T cells from IBV vs. IAV are shown at right.

**Figure 8:**
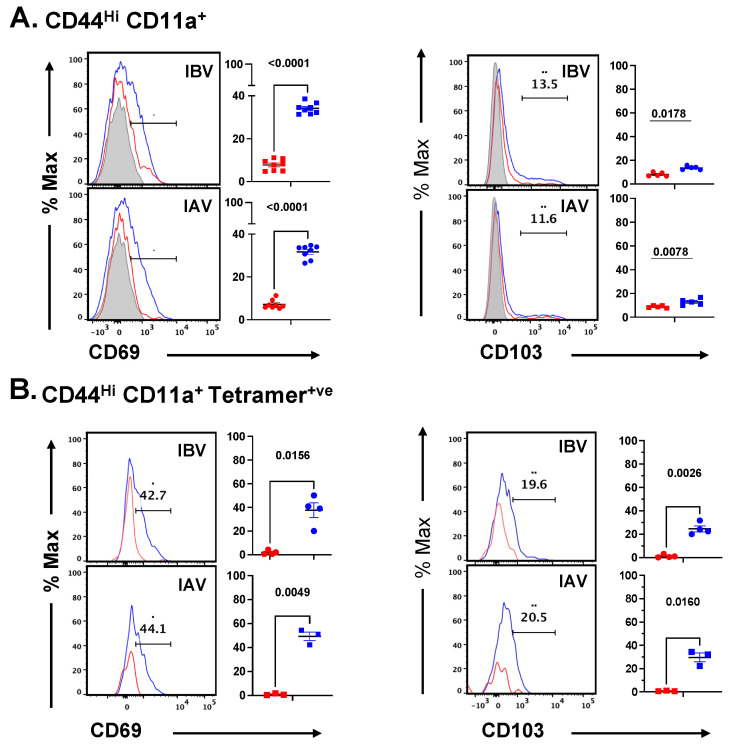
Lung distribution and cell surface phenotype of influenza-elicited CD4 T cells quantified by cell surface markers vs. MHC class II:peptide tetramers. Gating schemes used to identify and quantify CD4 T cells for the expression of CD69 and CD103 are shown in Supplemental Figure S1 and Supplemental Figure S3A,B. The negative control indicated in gray represents the pattern observed with FMO controls (see Section 4).

Previous studies of seasonal H1N1 and experimental strains of mouse adapted IAV [79,81] have shown that lung vasculature and lung tissue-localized CD4 T cells have distinctive cell surface phenotypes, which is in agreement with other studies of lung tissue T cells [37,81,82,86,87]. These have not been analyzed in IBV-infected mice. One critical adhesion molecule involved in extravasation, LFA-1, binds to intercellular adhesion molecule-1 (ICAM-1), which is up-regulated by inflammation expressed on vascular endothelium. As T cells access this environment, LFA-1 becomes activated and promotes higher affinity and more stable interactions with ICAM. The cell surface density of LFA-1 increases as the cells localize to the inflamed tissue. CD69 has been defined as a prototypic marker associated with the tissue residence of T cells, during both acute infections and at memory. Cytokines and local antigen are both thought to induce CD69 expression, which serves to enhance the tissue localization of effector T cells in part through reciprocal antagonism of the S1P receptor [88,89]. Thus, these molecules are thought to reflect important events in extravasation and lung residency. We assessed the expression of these prototypical markers of tissue residence on CD4 T cells localized to the lung tissue and vasculature in the IBV-infected mice at the peak of the response. As before, IAV-infected mice were examined in parallel. The gating strategy of lung T cells isolated from the infected mice is shown, with the populations selected, in Supplemental Figure S2. Data from flow cytometry using replicate mice are shown, with CD69 shown in Figure 6 (and later quantified in Figure 8) and CD11a in Figure 7A. In lungs of both IBV- and IAV-infected mice, analyses of tissue-localized CD4 T cells (in blue, unstained by IV administered antibody) relative to vasculature-localized CD4 T cells (in red) indicate that approximately half of the antigen-experienced cells express CD69, and the majority express high levels of CD11a. These results are similar to studies exploring lung-resident CD4 T cells from mice infected with the mouse adapted H1N1 (A/PR/8/34) [81] and the prior seasonal H1N1 strain (A/New Caledonia 20/99 [79]). The source of heterogeneity in CD69 expression in lung resident T cells observed in many studies after influenza infection has not yet been defined. CD69 negative T cells have been found at memory [84,90], arguing that these cells do not reflect kinetic intermediates. We speculate here that this heterogeneity may reflect the localization of T cells in lung sub-compartments that are rich in cytokines or antigen-bearing cells. Figure 7 quantifies the pattern of surface expression of CD11a, which is a key molecule for the extravasation of T cells from the lung vasculature to the tissue. In both IBV (top) and IAV (bottom)-infected mice, most of the antigen-experienced CD4 T cells extravasate to the lung tissue and display high levels of surface density of CD11a. Collectively, from these experiments, we conclude that IBV as well as IAV elicit CD4 T cells that home to the lung tissue and express these prototypic markers of tissue residency.

MHC class II peptide tetramers are powerful tools to isolate and query the cell surface phenotype, persistence, differentiation, and transcriptome of single-epitope specific CD4 and CD8 T cells (reviewed in [91,92,93,94,95]). However, because of potential idiosyncratic features of particular peptide: MHC-TcR complexes, there are advantages to assess the features of all the elicited T cells and include these in analyses of T cells that are specific for all peptides, including minor epitopes and those that have a broad range of T cell receptor affinity [96,97]. To examine whether the surface phenotype and distribution of CD4 T cells identified by the expression of high levels of CD44 and CD11a (see gating strategy in Supplemental Figure S2) agree with that determined by tracking epitope-specific CD4 T cells, we directly compared assessment of the distribution of influenza-specific CD4 T cells in the lung vasculature vs. tissue and the expression of markers associated with tissue residency. Accordingly, I-A^b^-peptide tetramers were derived to major epitopes in influenza B and influenza A. After preliminary testing, MHC class II: peptide tetramers were used to isolate CD4 T cells of the defined antigen specificity (Figure 8). A protocol developed by Jenkins et al. [28], using pre-enrichment of tetramer-positive cells from CD4 T cell-enriched populations from the lung, using magnetic columns before further analyses in order to eliminate the majority of non-antigen specific CD4 T cells was used. Then, the expression of cell surface markers on the lung cells isolated from IBV vs. IAV infected cells, which were identified as being in the lung vasculature or lung tissue as described above, was assessed on the tetramer positive cells. The flow cytometry scheme for IBV and IAV tetramer-positive cells is shown in Supplemental Figure S3A,B, respectively. The data using the tetramers were compared with that obtained from the polyclonal population identified using CD44 and CD11 bright cells (see Supplementary Figure S2) and then compared for the expression of surface markers associated with tissue residence. The influenza B-virus specific CD4 T cells partition into the lung, with the majority of cells at day 10 post infection residing in the lung tissue. Within the tissue, many of the tetramer-positive cells express CD69 and high levels of CD11a. A small fraction of cells express CD103. These data, collectively using epitope-specific tracking and cell surface markers of tissue resident cells, emphasize the value of utilizing CD44 and CD11a to analyze the polyclonal population of CD4 T cells. This approach complements the insight obtained from tracking CD4 T cells of a single epitope. These experiments enable us to conclude that both the polyclonal virus-specific CD4 T cells that express diverse antigen specificity and epitope-specific CD4 T cells elicited by IBV infection are primarily localized to the lung tissue and express several markers associated with tissue residency at the site of infection.

## 3. Discussion

As a result of the critical role that CD4 T cells play in protective immunity to influenza and other respiratory pathogens, the ability to track and analyze the specific CD4 T cell responses to infection or responses after vaccination to influenza virus challenge is critical. There has been little progress thus far in tracking CD4 T cells to influenza B viruses or vaccines, and little was known regarding epitope diversity, lung tissue localization, or the compartmentalization of CD4 T cells elicited by IBV (see reviews 39, 40, and 42). In this study, we filled this gap in knowledge and capabilities by developing a C57BL/6 model system to study CD4 T cell responses to influenza B/Brisbane/60/2008. Our studies have revealed that this Victoria lineage virus, isolated from human subjects, replicates and elicits a robust CD4 T cell response after intranasal infection. These analyses have indicated that the CD4 T cells elicited by IBV infection are quite diverse, and at the peak of infection, IBV-specific CD4 T cells are distributed widely across the secondary lymphoid tissues in the periphery. The tracking of virus-specific CD4 T cells into the lung post-infection, using both MHC class II: peptide tetramers and flow cytometry to identify the cells elicited by infection with IBV, indicate that these IBV-elicited cells in the lung express prototypic cell surface markers of tissue residency.

Influenza B has been relatively understudied in the field, in part because of the limited concern regarding pandemic potential, due to its current lack of an established animal reservoir. However, these viruses are now understood to cause significant morbidity and mortality, particularly in children, accounting for almost 25% of influenza infections each year and over 50% of influenza-related deaths in children (reviewed in [39,41,42,98]). For these reasons, there is increasing attention to influenza B vaccines, which have become quadrivalent in recent years to accommodate the two different lineages of influenza B that tend to co-circulate [99,100], as well as the development of novel vaccine candidates for influenza B [101,102,103] and novel therapeutic reagents [104,105,106]. The studies here are important for future clinical studies relevant to human disease. Often, the first step in developing novel vaccines and anti-viral reagents is the establishment of preclinical mouse models that allow rapid and well-controlled analyses of efficacy. There have been a number of recent studies of this type [43,101,102,103,104,105,106,107,108,109]. However, there have been limited analyses of the impact on the antigen-specific adaptive responses and in particular CD4 T cell responses, which are the critical regulators of both B cell and CD8 T cell immunity. Beyond enabling more mechanistic studies of vaccines and anti-viral reagents, the studies presented here in B6 mice can also enable the mapping of human CD4 T cell epitopes using HLA-transgenic mice, which are most often generated on the B6 background, due to the availability of the murine class II deficient strain of B6, which is available through commercial sources. Our laboratory has defined a number of IAV-derived CD4 T cell epitopes using HLA-transgenic mice of this type [110]. Other laboratories have contributed to this knowledge as well [111,112,113]. Enhanced CD4 T cell epitope discovery for IBV in HLA-DR and DQ transgenic mice will enable more sophisticated epitope-specific tracking of human CD4 T cells from the polyclonal population through the derivation and implementation of HLA-peptide tetramer reagents (reviewed in [92,93,114]). The studies presented here are expected to promote a more comprehensive understanding of the events in the adaptive response to vaccination and infection by IBV viruses and vaccine candidates, thus complementing the more extensive and accumulating data on IAV. Collectively, the advancement of human clinical studies will be accomplished through an array of preclinical studies of vaccines and anti-viral reagents. The use of conventional B6 mice for these studies as well as in transgenic and gene deletion models should provide more insight into the unique features of the host immune response to IBV infection and specifically the role that host CD4 T cell responses play in protection from infection and the benefits of vaccination to establishing long-term protective immunity in both animal model and in human studies.

## 4. Materials and Methods

### 4.1. Ethics Statement

All mice were maintained in a specific-pathogen-free facility at the University of Rochester Medical Center according to institutional guidelines. All animal protocols adhere to the AAALAC International, the Animal Welfare Act, and the PHS Guide, and they were approved by the University of Rochester Committee on Animal Resources, Animal Welfare Assurance Number A3291-01. The protocol under which these studies were conducted was originally approved 4 March 2006 (protocol no. 2006-030) and has been reviewed and re-approved every 36 months with the most recent review and approval in 29 December 2020.

### 4.2. Mice and Viruses

C57BL/6 female mice were purchased from NCI, and B10.S and B10 male and female mice were purchased from Jackson Laboratories and maintained in our laboratory. All mice were used at 2–5 months of age and were maintained at the University of Rochester according to institutional guidelines. Influenza A virus (A/California/2009/E3) virus was produced as we have previously described [115,116]. Influenza B virus (B/Brisbane/60/2006) was produced in eggs and quantified as described [60,117].

### 4.3. Peptides

In total, 16 to 17-mer peptides overlapping by 11 amino acids to encompass the entire sequence of the HA, NA, NP, M1, and NS1 proteins from each influenza virus, A/California/04/09, and B/Brisbane/60/2008 were obtained from BEI Resources, ATCC. Influenza B NS1 peptides were purchased from Mimotopes. Supplemental Table S1 provides the sequences and nomenclature for the peptides used in these studies.

### 4.4. Virus Infections

C57BL/6 mice received infectious virus at a dose of 4800 PFUs of B/Brisbane/60/2008 and 300 PFUs of A/California/07/2009. B10.S and B10 mice were infected at 3800 PFU of B/Brisbane/60/2008 and 240 PFU of A/California/07/2009. Mice were anesthetized by intra-peritoneal injection with tribromoethanol (Avertin 14 μL/mg body weight), and virus, diluted in 30 μL of phosphate-buffered saline (PBS), was instilled intranasally. At indicated time points post-infection, mice were euthanized, and the lungs, mLN, and spleen were excised and processed into single-cell suspension as previously described [62] for cellular assays or flow cytometry.

### 4.5. Lung Virus Titers and TCID_50_

For lung virus titration, lungs were harvested under sterile conditions at D2 and D6 post-infection and stored at −80 °C. Samples were thawed and homogenized by mechanical homogenization using a glass homogenizer, as previously described [118,119], and the suspensions were clarified by centrifugation at 2000× *g* for 10 min. Virus titers were determined by TCID_50_ assay in MDCK cells. MDCK monolayers were inoculated with 100 μL of lung homogenate dilutions from 10^−1^ to 10^−8^ in triplicates. Influenza A virus was incubated at 37 °C, and influenza B virus was incubated at 33 °C with 5% CO_2_ for 72 h. The endpoint is determined by HA titer with freshly prepared 0.5% packed chicken erythrocytes. The titer is calculated using the method of Reed and Muench and expressed as the mean log10TCID_50_/_mL_ ± the standard deviation (SD) [120].

### 4.6. EliSpot Assays

EliSpot assays to detect cytokine-producing cells were performed as previously described [64,80,118]. Briefly, 96-well filter plates were coated with 2 μg/mL purified rat anti-mouse IL-2 or IFNγ (clone JES6-1A12, clone AN-18, respectively, BD Biosciences) in PBS overnight at 4 °C, washed with media to remove any unbound antibody, and incubated with 100 μL media per well for 1 h to block non-specific binding. Isolated CD4 T cells, plated at the optimal concentration to enumerate cells (20,000 to 50,000 per well for lung, 100,000–250,000 for mLN, and 300,000 for splenocytes) were co-cultured with 500,000 syngeneic spleen cells and peptide at a final concentration of 5 μM in a total volume of 200 μL for 18–20 h at 37 °C and 5% CO_2_. The cells were removed from the plates, and the plates were washed with wash buffer (1X PBS, 0.1% Tween-20). Biotinylated rat anti-mouse IL-2 or IFNγ (clone JES6-5H4, clone XMG1.2, respectively, BD Biosciences) was added at a concentration of 2 μg/mL, 50 μL/well, in wash buffer with 10% FBS and incubated at room temperature for 30 min. The plates were washed again, and streptavidin-conjugated alkaline phosphatase (Jackson ImmunoResearch) was added at a dilution of 1:1000 in wash buffer with 10% FBS, 50 μL well, and incubated for 30 min at room temperature. The plates were washed with wash buffer and developed using Vector Blue substrate kit III (Vector Laboratories) prepared in 100 mM Tris, pH 8.2. After drying, quantification of spots was performed with an Immunospot reader series 5.2, using Immunospot software, version 5.1. Results are presented as the mean ± standard error of the mean (SEM) or range of the response, with background subtracted. Statistical analyses were performed using GraphPad software version 5.0a.

### 4.7. IV labeling, Tetramer Enrichment, and Flow Cytometry

As previously described [80], mice were injected intravenously with APC-CD45 antibody prior to sacrifice to mark CD4 T cells in the lung vasculature. Briefly, after labeling cells in vivo for 3 min with CD45-APC, lungs were harvested and processed into single-cell suspensions. A cocktail of antibodies, titrated to optimal concentration, was used to resolve surface markers on CD4 T cells and included CD19 (1D3, BD Horizon), CD4 (RAM4-5, BD Pharmigen), CD8a (53-6.7, Biolegend), TCRβ (H57-597, Biolegend), CD44 (IM7, Tonbo), CD62L (MEL-14, Biolegend), CD11a (2D7, BD Biosciences), CD69 (H1.2F3, Biolegend), and CD103 (2E7, Biolegend). For tetramer staining, PE-conjugated tetramer with the peptide indicated in the figure legends with the above-mentioned CD4 T cells markers were used to stain virus-specific CD4 T cells. Prior to staining, tetramer-specific CD4 T cells were enriched using negative MACS enrichment [62]. Data were acquired using a BD LSR-II instrument, configured with 488 (blue), 633 (red), 407 (violet), and 532 (green) nm lasers, as previously described [26]. Data were analyzed using Flowjo software (Flowjo, LLC.), version 10.

## Data Availability

The full complement of data accumulated for these studies is available upon request.

## Supplementary Materials

The following supporting information can be downloaded at: https://www.mdpi.com/article/10.3390/pathogens11020251/s1, Figure S1: Gating scheme to quantify naïve (CD44^−^ CD62L^+^) and CD44^hi^ CD4 T cells in uninfected mice; Figure S2: Gating scheme for analyses of polyclonal CD4 T cells in lung; Figure S3: Flow cytometry gating scheme using MHC class II: peptide tetramers; Table S1: Peptide nomenclature and sequences; Table S2: IFNγ EliSpot kinetics of CD4 T cell response in different tissues.
