# Supplementary material for: Development of a Mouse Model to Explore CD4 T Cell Specificity, Phenotype, and Recruitment to the Lung after Influenza B Infection
Source: Pathogens. 2022 Feb 15;11(2):251. doi: 10.3390/pathogens11020251 (PMC8875387; doi:10.3390/pathogens11020251)
Supplement: Supplementary file 1 [file pathogens-11-00251-s001.zip › supplementary material conversion.pdf]

Supplemental Data

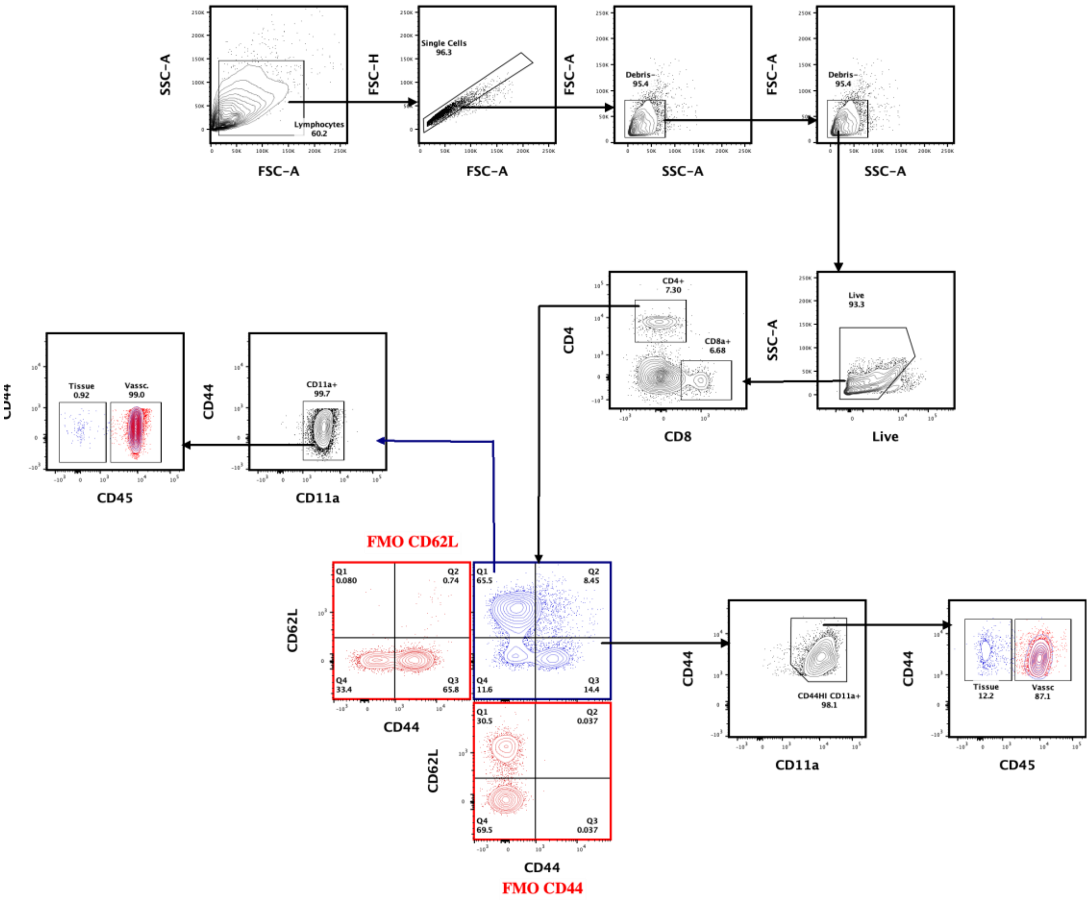

Supplementary Figure S1. Gating scheme to quantify naïve ($CD44^{-}$ $CD62L^{+}$) and $CD44^{hi}$ CD4 T cells in uninfected mice shown for data in Figure 5.

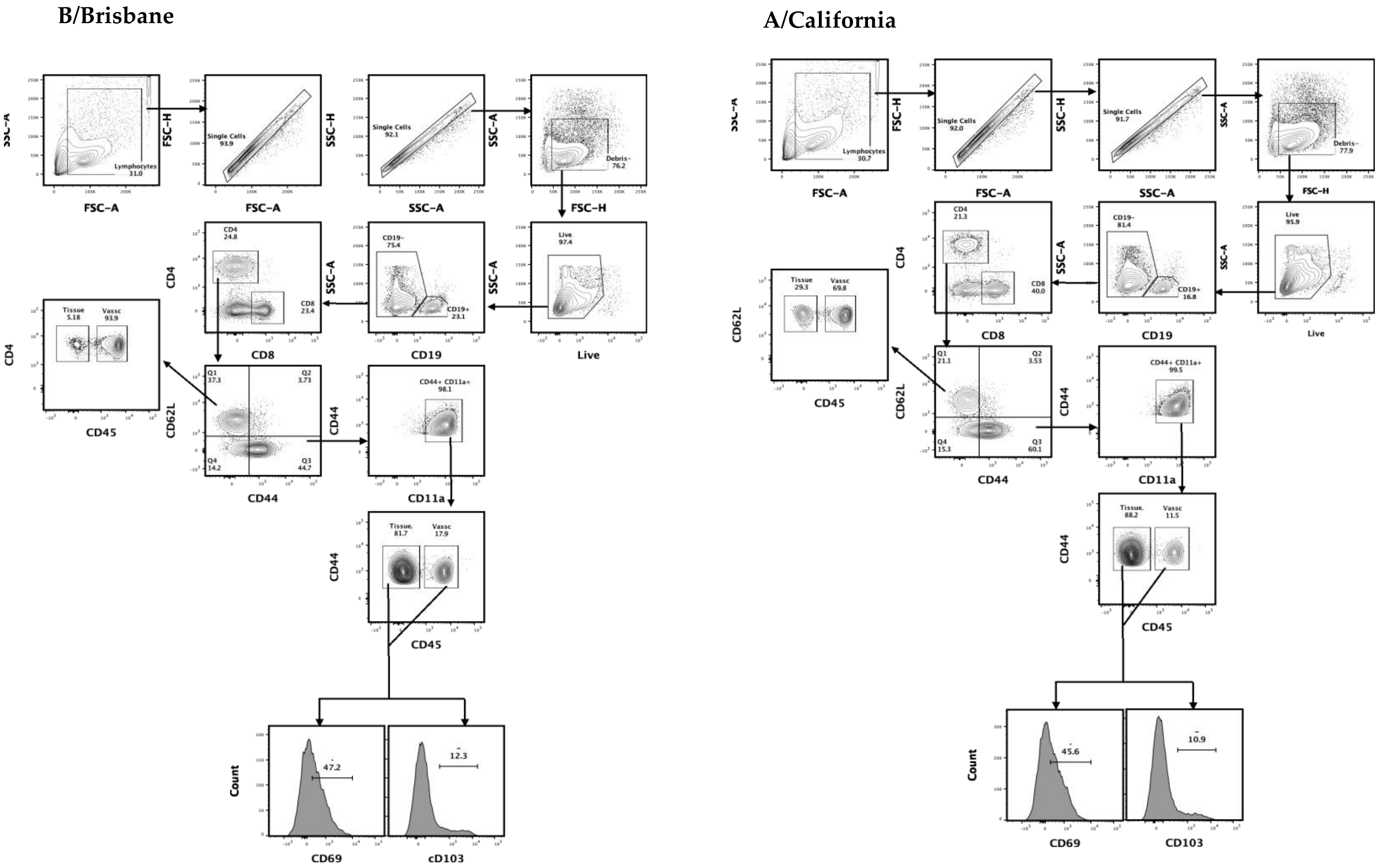

Supplemental Figure S2. Gating scheme for analyses of polyclonal CD4 T cells in lung. A sequential gating scheme as shown was used to identify antigen experienced CD4 T cells in the lung vasculature and tissue and to quantify surface markers (CD11a, CD69 and CD103, as shown).

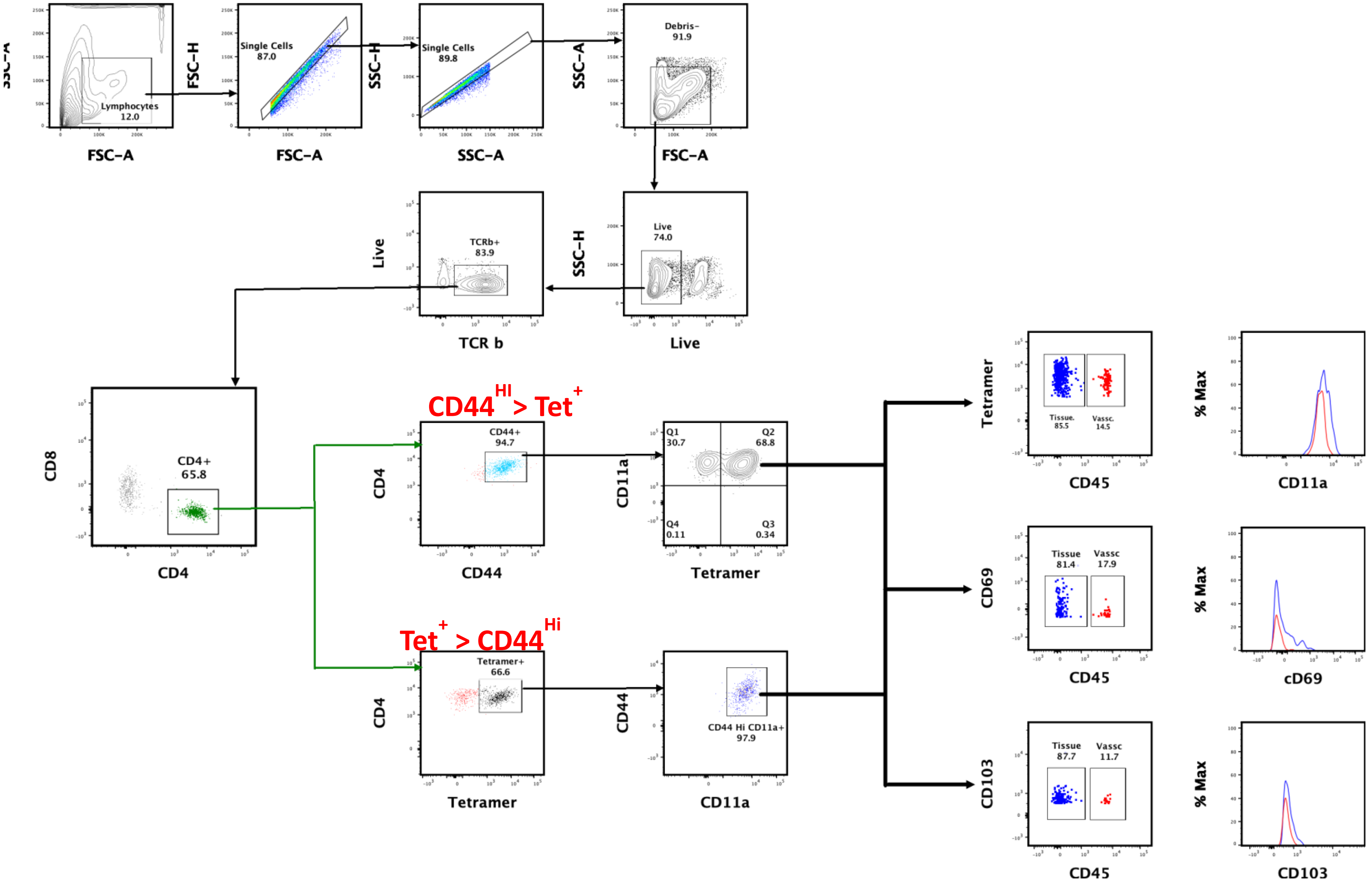

Supplementary Figure S3A. Flow cytometry gating scheme using MHC class II: peptide tetramers from lung CD4 T cells that were isolated from influenza B infected mice. Shown is the sequential gating scheme used to identify peptide epitope-specific CD4 T cells using the I-A^{b}:HA-23 peptide tetramer (see Supplemental Table S1).

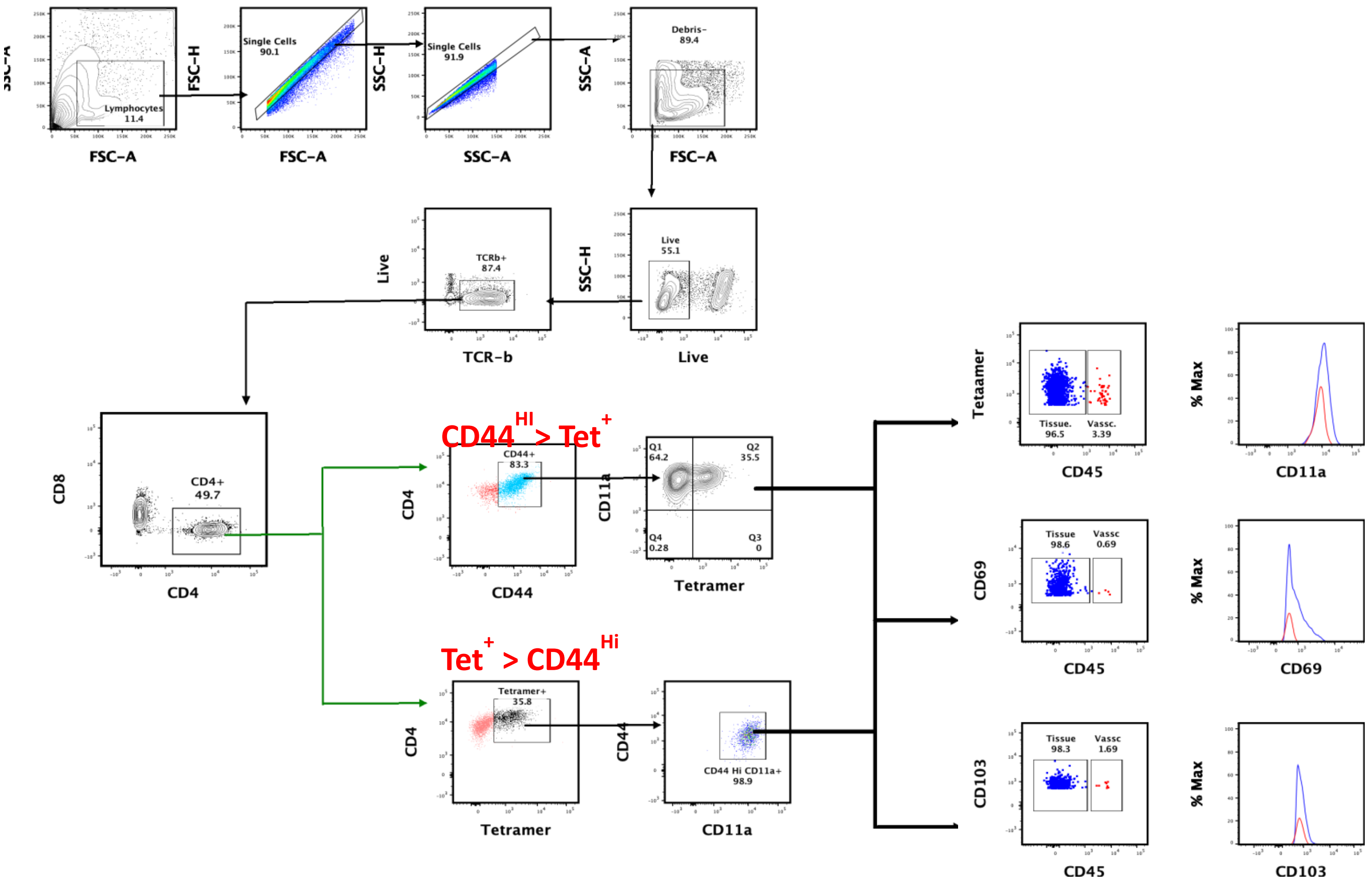

Supplementary Figure S3B. Flow cytometry gating scheme using MHC class II: peptide tetramers from lung CD4 T cells isolated from influenza A infected mice. Shown is the sequential gating scheme used to identify peptide epitope-specific CD4 T cells using the I-A^{b}:NP-263 peptide tetramer (see Supplemental Table S1).

Supplemental Table S1. Peptide nomenclature and sequences.

TABLE S1.1: Influenza B peptides

Peptide name	Peptide sequence	Peptide length
*HA 23	**23 TSSNSPHVVKTATQGE 38**	16
HA 97	**97 SILHEVRPVTSGCFP 111**	15
HA 483	**483 KLKKMLGPSAVEIGN 497**	15
NP 316	**316 LLARSMVVVRPSVASKV 332**	17
NP 321	**321 MVVVRPSVASKVVLPIS 337**	17
NP 346	**346 GFNVEEYSMVGYEAMAL 362**	17
NP 358	**358 EAMALYNMATPVSILRM 374**	17
NP 391	**391 GAAYEDLRVLSALTGTE 407**	17
NP 465	**465 SPVFAVERPIALSKQA 480**	16
NA 77	**77 EPEWTYPRLSCPGST 91**	15
NA 173	**173 HMAAWSGSACHDGKE 187**	15
NA 177	**177 WSGSACHDGKEWTYI 191**	15
NA 321	**321 TYLDTPRPNDGSITG 335**	16
NS1 8	**8 TTQIEVGPGATNATIN 22**	16
NS1 85	**85 MKVLLFMNPSAGIEGF 100**	16
NS1 89	**89 LFMNPSAGIEGFEPYC 104**	16
NS1 109	**189 VNGTFLKHPNGYKSLS 204**	16

TABLE S1.2: Influenza A/California peptides

NA 28	NA 109	**109-GSKGDVFVIREPFIS-123**	15
NA 39	NA 153	**153-SPYRTLMSCPIGEVPS-168**	16
NA 45	NA 177	**177-VAWSASACHDGINWL-191**	15
NA 56	NA 221	**11515221-NNILRTQESECACVN-235**	15
NS1 3	NS1 9	**9-FQVDCFLWHIRKRFA-23**	16
NS1 4	NS1 13	**13-CFLWHIRKRFADNGL-27**	16
NS1 13	NS1 49	**49-TLGLDIETATLVGKQ-63**	16
NS1 23	NS1 89	**89-YLSDMTLEEMSRDWF-103**	16
M1 35	M1 137	**137-TVTTEAAFGLVCATC-151**	15
M1 46	M1 181	**181-LASTTAKAMEQMAGS-195**	15
M1 47	M1 185	**185-TAKAMEQMAGSSEQA-199**	15
M1 53	M1 209	**209-TRQMVHAMRTIGTHPS-224**	15
M1 55	M1 217	**217-RTIGTHPSSSAGLKD-231**	15

Supplemental Table S2. IFNγ EliSpot kinetics of CD4 T cell response in different tissues.

Virus	B/Brisbane CD4 T cells Spots/million				A/California CD4 T cells Spots/million		
Viral Protein	Day 7	Day 10	Day 16	Viral protein	Day 7	Day 10	Day 16
**Lung**							
**NA-B**	3490.0	11727.5	7628.5	**NA-A**	932.5	8080.0	6725.0
**NP-B**	2575.8	11715.0	7029.9	**NP-A**	3541.7	9082.5	7050.0
**mLN**							
**NA-B**	1535.8	2156.1	2138.8	**NA-A**	131.7	986.3	385.8
**NP-B**	1361.7	2693.3	2297.9	**NP-A**	322.3	1051.7	652.1
**Spleen**							
**NA-B**	2478.9	1670.6	1142.9	**NA-A**	1251.0	445.6	82.5
**NP-B**	2406.2	2115.6	1423.8	**NP-A**	2293.5	613.1	181.9
